# The Effect of Intraocular Pressure Load Boundary on the Biomechanics of the Human Conventional Aqueous Outflow Pathway

**DOI:** 10.3390/bioengineering9110672

**Published:** 2022-11-10

**Authors:** Alireza Karimi, Reza Razaghi, Seyed Mohammadali Rahmati, J. Crawford Downs, Ted S. Acott, Mary J. Kelley, Ruikang K. Wang, Murray Johnstone

**Affiliations:** 1 Department of Ophthalmology and Visual Sciences, University of Alabama at Birmingham, Birmingham, AL 35233, USA; 2 School of Biological Sciences, Georgia Institute of Technology, Atlanta, GA 30332, USA; 3 Departments of Ophthalmology and Biochemistry and Molecular Biology, Casey Eye Institute, Oregon Health & Science University, Portland, OR 97239, USA; 4 Departments of Ophthalmology and Integrative Biosciences, Casey Eye Institute, Oregon Health & Science University, Portland, OR 97239, USA; 5 Department of Ophthalmology, University of Washington, Seattle, WA 98195, USA; 6 Department of Bioengineering, University of Washington, Seattle, WA 98195, USA

**Keywords:** trabecular meshwork, juxtacanalicular tissue, Schlemm’s canal, viscoelastic material model, transient IOP fluctuations, fluid–structure interaction

## Abstract

Background: Aqueous humor outflow resistance in the trabecular meshwork (TM), juxtacanalicular connective tissue (JCT), and Schlemm’s canal (SC) endothelium of the conventional outflow pathway actively contribute to intraocular pressure (IOP) regulation. Outflow resistance is actively affected by the dynamic outflow pressure gradient across the TM, JCT, and SC inner wall tissues. The resistance effect implies the presence of a fluid–structure interaction (FSI) coupling between the outflow tissues and the aqueous humor. However, the biomechanical interactions between viscoelastic outflow tissues and aqueous humor dynamics are largely unknown. Methods: A 3D microstructural finite element (FE) model of a healthy human eye TM/JCT/SC complex was constructed with elastic and viscoelastic material properties for the bulk extracellular matrix and embedded elastic cable elements. The FE models were subjected to both idealized and a physiologic IOP load boundary using the FSI method. Results: The elastic material model for both the idealized and physiologic IOP load boundary at equal IOPs showed similar stresses and strains in the outflow tissues as well as pressure in the aqueous humor. However, outflow tissues with viscoelastic material properties were sensitive to the IOP load rate, resulting in different mechanical and hydrodynamic responses in the tissues and aqueous humor. Conclusions: Transient IOP fluctuations may cause a relatively large IOP difference of ~20 mmHg in a very short time frame of ~0.1 s, resulting in a rate stiffening in the outflow tissues. Rate stiffening reduces strains and causes a rate-dependent pressure gradient across the outflow tissues. Thus, the results suggest it is necessary to use a viscoelastic material model in outflow tissues that includes the important role of IOP load rate.

## 1. Introduction

The aqueous humor outflow resistance in the conventional outflow pathway is the primary determinant of intraocular pressure (IOP) [1,2,3,4]. Dysregulation in the balance between the aqueous inflow and outflow can result in an IOP elevation that is associated with primary open-angle glaucoma (POAG) [5,6,7,8,9,10,11,12]. Aqueous humor passes through the trabecular meshwork (TM) and the juxtacanalicular connective tissue (JCT). After crossing the TM, JCT, and inner wall endothelium of Schlemm’s canal (SC), aqueous humor enters the SC lumen and eventually flows circumferentially to the collector channels leading to the aqueous and episcleral veins [13,14,15,16,17].

Transient IOP fluctuations [12,18,19,20,21,22,23,24] result in a dynamic mechanical environment in the outflow pathway that actively affects the geometry and cellular mechanotransduction of the outflow tissues [25,26,27] as well as the outflow resistance [28]. Active outflow resistance regulation in the conventional outflow pathway results in a dynamic outflow pressure gradient across the outflow tissues [9,29,30]. The TM responds mechanically to IOP fluctuations by undergoing geometric changes, resulting in an interaction between the outflow tissues and aqueous humor [29]. Cell and tissue constituents of the TM, JCT, and SC respond to the pressure gradient by adjusting the tissue and cellular elasticity [17]. Thus, TM/JCT/SC motion and its resulting mechanisms of aqueous outflow resistance [31,32] mainly depend on the biomechanical properties of outflow tissues [33,34,35,36]. The pulsatile motion of the TM reflects the tissue’s deformability in response to the cardiac-induced ocular pulse amplitude [37], and the TM’s stiffness is a key parameter in determining TM pulsatile motion [38]. It has been shown that the TM experiences different displacements with IOP fluctuation [31,32] that demonstrate the viscoelasticity of the TM tissue. However, one may argue that these displacements in the TM relate to the ocular pulse since a significant correlation has been reported between the TM displacement and ocular pulse amplitude [31]. However, Li and colleagues used phase-sensitive optical coherence tomography (PhS-OCT) to show that TM displacement is strongly IOP load rate-dependent at a constant ocular pulse amplitude in ex vivo nonhuman primate eyes (*Macaca nemestrina*). Thus, as load rate increases, the same IOP pulse amplitude results in progressively smaller displacements of the TM [33], indicating that the TM is viscoelastic.

To date, experimental [38,39,40] and numerical [40,41] TM biomechanics studies have been limited to using isotropic elastic and hyperelastic mechanical properties to model the TM, and yet soft biological tissues are generally both anisotropic and viscoelastic [42,43,44,45,46,47]. Elastic materials restore the specimen to its initial configuration by releasing stored energy from loading. In contrast, viscoelastic materials dissipate some stored energy [48], causing a load rate-dependent mechanical response. IOP causes microscopic mechanical deformations in the extracellular matrix of the TM, JCT, and SC inner wall tissues [27,49,50,51]. Soft biological tissues such as those in the outflow pathway are typically viscoelastic and their mechanical responses depend on the load rate. Therefore, it is reasonable to speculate that the outflow pressure gradient across the TM/JCT/SC inner wall complex depends on the rate of change in IOP (load), which may result in dynamic changes in outflow resistance. However, the dynamics of aqueous humor interactions with the local and global biomechanical responses of the outflow pathway tissues are largely unknown.

Karimi and colleagues recently calculated the viscoelastic mechanical properties of the TM/JCT/SC complex [52] using a finite element (FE)-optimization method and dynamic SC pressurization experimental setup [53]. We showed that the elastic material model was unable to capture the time-dependent mechanical response of the outflow tissues. In contrast, the viscoelastic material model successfully captured the tissues’ dynamic motion and resulted in a good match with spectral domain PhS-OCT imaging data [53]. Applying the viscoelastic material model allows us to include the important effect of dynamic IOP changes across the outflow pathway. The model can then simultaneously assess the hydrodynamics and the resultant stresses and strains of the outflow tissues [48,54,55,56]. The findings may significantly enhance the accuracy of the modeling results. It is suggested that there must be an active fluid–structure interaction (FSI) providing coupling between the outflow tissues and outflow resistance that actively contributes to IOP regulation [57,58]. Experimental studies also showed a significant correlation between cyclic responses that affect the biomechanics of the outflow tissues and the resultant balance of aqueous inflow and outflow [59]. Doubling the inflow rate resulted in an immediate ~2-fold IOP elevation, which returned to baseline regulation after several days of continued perfusion [57].

Karimi [60] recently developed a 3D FSI microstructural model of a healthy human conventional outflow pathway. We showed a larger aqueous humor pressure drop across the outflow pathway with stiffer tissues. However, we used a simple elastic material model with an idealized IOP load boundary that may not fully represent the dynamic biomechanical behavior seen with a physiologic load boundary. While great strides have been made in understanding the mechanisms that regulate aqueous outflow resistance in the conventional outflow pathway [3,4,28,58,61,62,63,64,65], the mechanism of outflow resistance regulation with a dynamic physiologic IOP load boundary is largely unknown. Although it has been shown experimentally [38,66] and numerically [60] that increased TM stiffness causes increased outflow resistance, it remains unclear how viscoelastic outflow tissues with a physiologic IOP load boundary may affect outflow resistance. To date, clinical and experimental techniques have been unable to quantify the biomechanical stresses and strains in the outflow tissues considering their time-dependent mechanical properties and transient IOP fluctuations. Numerical approaches, such as the FSI, allow us to calculate the local resultant stresses and strains across the TM, JCT, and SC inner wall tissues, as well as hydrodynamics of the aqueous humor. FSI studies can estimate the regions with relatively higher shear stresses that are thought to play an important role in IOP regulation through the endothelial nitric oxide pathway [67,68,69] and include the biomechanical interaction of aqueous humor with the deformable tissue walls that can advance our understanding of IOP regulation [19,27,53,60]. We can thereby conceptualize new approaches for diagnostic and therapeutic methods to cope with ocular hypertension and glaucoma [31].

In this study, we constructed a microstructural TM/JCT/SC complex FE model of a normal human eye [60] using the elastic and viscoelastic extracellular matrix of the outflow tissues with embedded elastic cable elements that represent the directional stiffness imparted by anisotropic collagen fibrils [53]. The tissues were subjected to an idealized and physiologic IOP load boundary by means of controlling steady state or pulsatile aqueous humor inflow. The resultant stresses and strains in the outflow tissues and hydrodynamics in the aqueous humor were calculated using the FSI method, and results were compared.

## 2. Materials and Methods

### 2.1. Human Eye Imaging, FE Reconstruction, Cable Elements Distribution, Material Models, Hydraulic Conductivity, and Boundary Conditions

**Figure 1:**
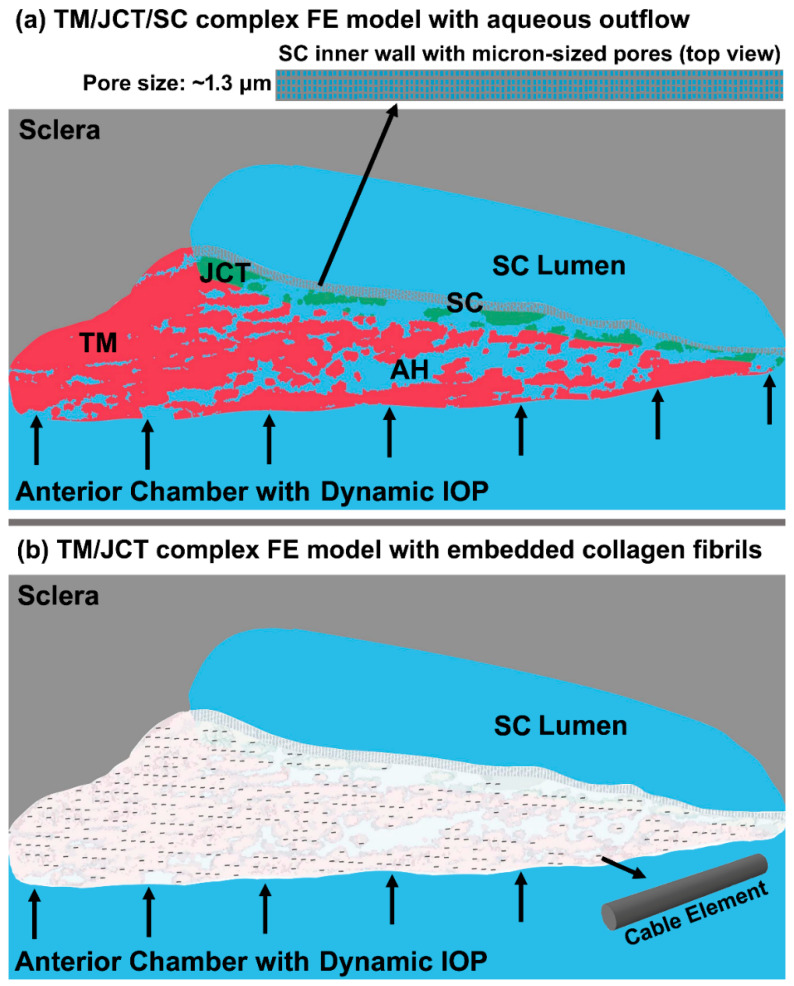
(**a**) The 3D microstructural FE model of the TM/JCT/SC complex and aqueous humor with (**b**) embedded elastic cable elements [60].

The FE model of the TM/JCT/SC complex consisting of the sclera, outflow system, and cornea was obtained as described in our prior publication [70]. The imaging, segmentation, and volume meshing methods of the TM/JCT/SC complex FE model were fully explained in our prior publications [53,60]. A wedge of the anterior segment was imaged and [71] volume meshed [70,72]. The model [73] was separated into the TM with adjacent JCT (~10 μm [71]) and SC inner wall (~5 μm [30]) regions as shown in Figure 1. Idealized µm-sized pores were distributed in the SC inner wall [74] with a pore density and diameter of 835 pores/mm^2^ [30] and 1.3 µm [75], respectively (Figure 1a inset). Element quality assessment was conducted using Ansys (Ansys Inc., Pittsburgh, PA, USA) [52,73,76,77,78] to make sure the Jacobian ratio, aspect ratio, warping factor, and skewness were within the acceptable ranges for a good quality element (Ansys Inc.). Mesh density analyses were performed for the FE model described in our prior publications [53,60].

The cable elements were distributed in the extracellular matrix of the TM and JCT (Figure 1b) using a mesh-free, penalty-based, cable-in-solid coupling algorithm [52] to represent the directional stiffness imparted by anisotropic collagen fibril orientation in those tissues [17,79,80,81,82,83,84,85,86]. The cable elements were modeled with an elastic material, and the extracellular matrix was modeled with elastic and viscoelastic materials using 8-noded hexahedral solid elements with a fully integrated element formulation [87]. The elastic modulus of the sclera was 2.93 MPa [88] and nearly incompressible (Poisson’s ratio, ν = 0.495) [89,90]. The elastic modulus of the TM/JCT/SC complex was 0.148 MPa, nearly incompressible (Poisson’s ratio, ν = 0.495), with the cable element elastic modulus of 1280 MPa as calculated in our prior publication using an FE-optimization algorithm matched with spectral domain PhS-OCT imaging data [53]. In the viscoelastic model, the material parameters were G_0_ (short-time shear modulus) = 6.36 MPa, G_∞_ (long-time shear modulus) = 1.08 MPa, *β* (decay constant) = 999.25 1/s, and cable element elastic modulus = 2814 MPa [53].

The hydraulic conductivities of 2.0 µL/min/mmHg/cm^2^ [91], 2.5 mmHg/µL/min/cm^2^ [92], and 9000 × 10^−11^ cm^2^ s/g [28] were programmed into the model for the TM, JCT, and SC inner wall extracellular matrix [60]. The TM, JCT, and SC inner walls were treated as tissues with the same mechanical properties but different hydraulic conductivities.

**Figure 2:**
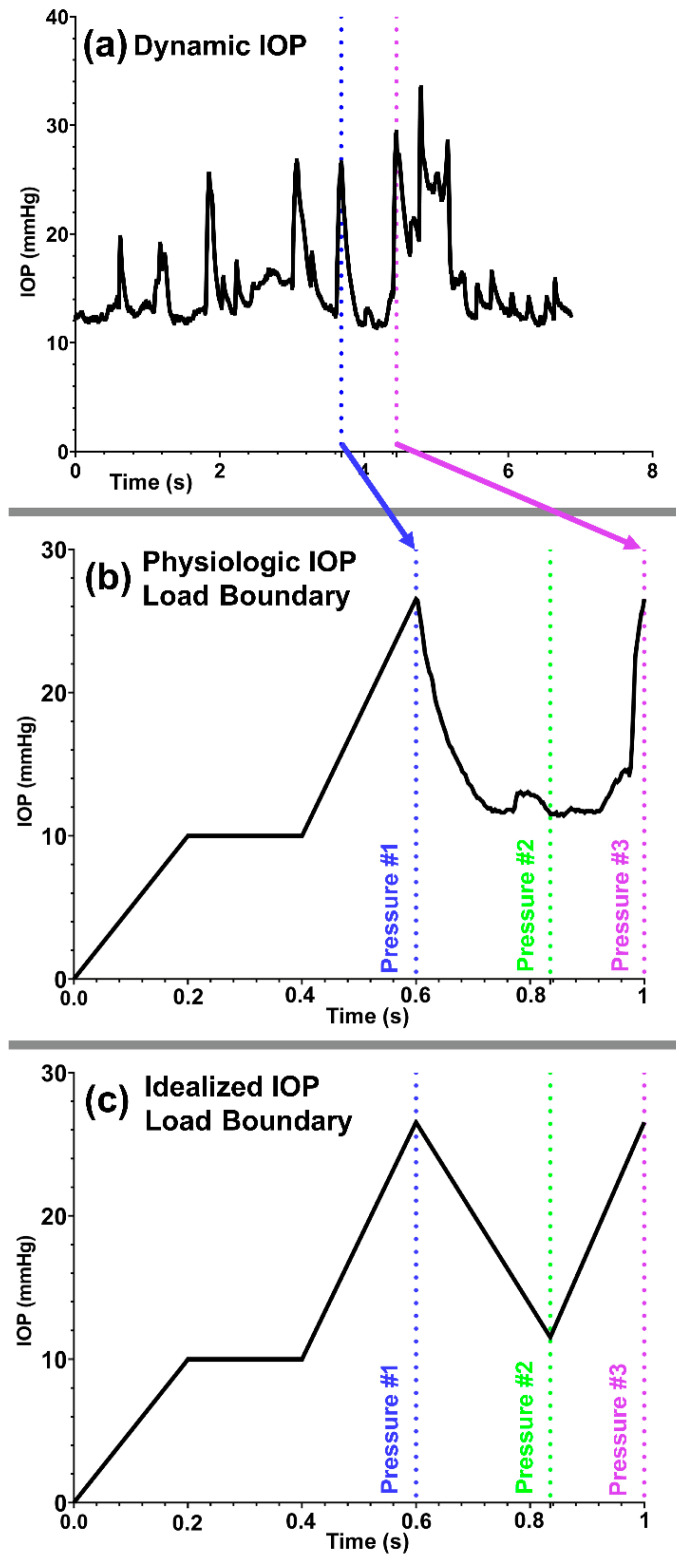
(**a**) Transient IOP fluctuations in a non-human primate (a rhesus macaque aged 4, male, right eye). (**b**) Physiologic and (**c**) idealized IOP load boundary with an IOP pre-load applied to the aqueous humor inflow.

A pre-tension force of ~500 μN [93] was induced in the TM/JCT/SC complex local nodes to mimic the ciliary muscle movement during IOP fluctuation [17], which also helps to prevent sudden excessive dynamic response in the cables [52]. Aqueous humor with a physiologic IOP load boundary adopted from a living non-human primate (Figure 2a,b) and an idealized IOP load boundary (Figure 2c) were flowed into the outflow pathway. Due to the limitation in our computational power, only a 400 ms range of physiologic IOP (Figure 2a) was selected and applied to the model (Figure 2b). 

### 2.2. Fluid–Structure Interaction

The FSI formulations were fully explained in our prior publication [60]. Briefly, the solid and fluid domains representing the TM/JCT/SC complex and aqueous humor were defined using an arbitrary Lagrangian–Eulerian (ALE) approach [94,95]. The multi material ALE (Ansys/LS-DYNA, Pittsburgh, PA, USA) automatic mesh refinement algorithm helped to enhance the modeling robustness and accuracy of Lagrangian and Eulerian mesh motions within the same framework, enhancing the modeling ability for curved surfaces of a complex geometry [96,97]. Aqueous humor was modeled as homogeneous, Newtonian, and viscous [98], with the density and dynamic viscosity of 1000 kg/m^3^ and 0.7185 mPa·s [99], respectively.

The pre-load was achieved by a linear IOP elevation to 10 mm Hg for 200 ms (time-step: 10 ms), then kept at 10 mmHg for 200 ms, and finally elevated to 26.54 mmHg for 200 ms. The pre-load was followed by a physiologic IOP load boundary for 400 ms (Figure 2b) [23,100]. An idealized IOP load boundary was also applied within 400 ms after the pre-loading to mimic the same maximum and minimum IOP magnitudes (Figure 2c) that occur in the physiological IOP load boundary (Figure 2b). An explicit dynamic solver was used to solve the problem. The elastic and viscoelastic FSI simulations on average took ~250 and ~436 h, respectively, to run on our workstation.

## 3. Results

**Figure 3:**
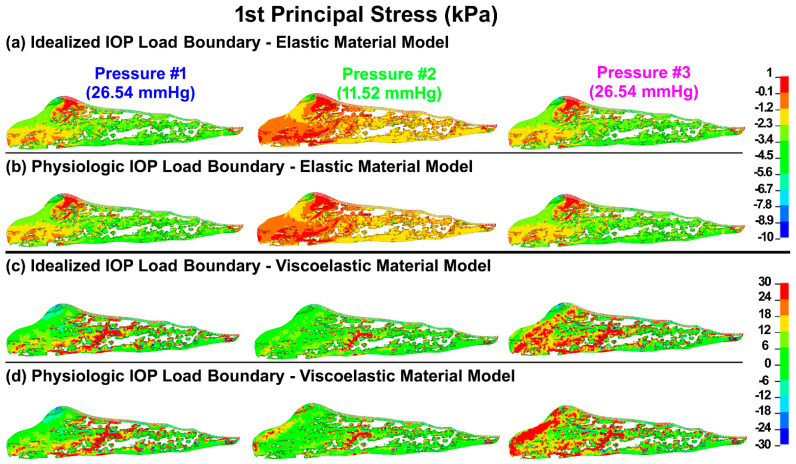
The 1st principal stress in the TM/JCT/SC complex with (**a**) idealized IOP load boundary (elastic material) (pressure t1, pressure t2, pressure t3), (**b**) physiologic IOP load boundary (elastic material), (**c**) idealized IOP load boundary (viscoelastic material), and (**d**) physiologic IOP load boundary (viscoelastic material) at three different IOPs.

**Figure 4:**
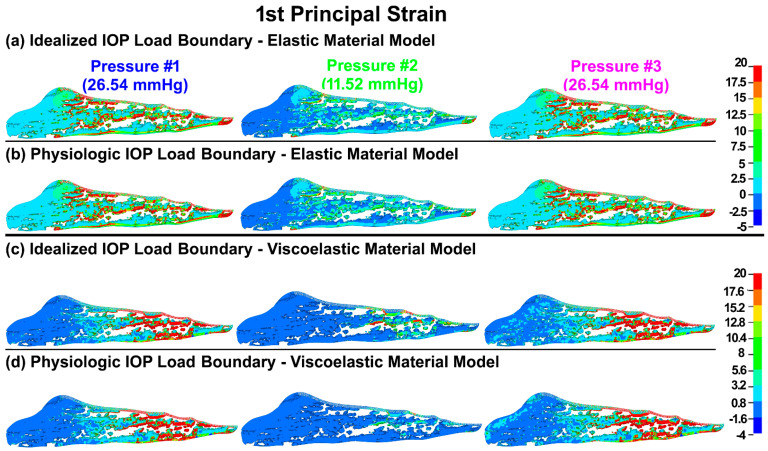
The 1st principal strain in the TM/JCT/SC complex with (**a**) idealized IOP load boundary (elastic material), (**b**) physiologic IOP load boundary (elastic material), (**c**) idealized IOP load boundary (viscoelastic material), and (**d**) physiologic IOP load boundary (viscoelastic material) at three different IOPs.

The first principal (tensile) stresses and strains in the TM/JCT/SC complex with idealized and physiologic IOP load boundary and elastic and viscoelastic material models at three different pressures are shown in Figure 3 and Figure 4, respectively.

**Figure 5:**
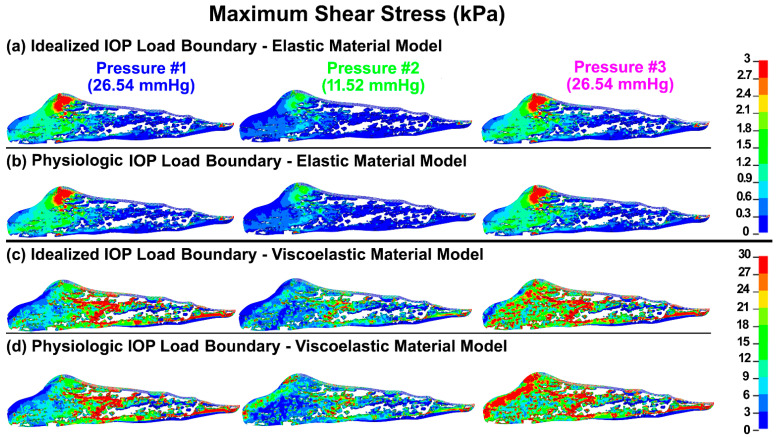
The maximum shear stress in the TM/JCT/SC complex with (**a**) idealized IOP load boundary (elastic material), (**b**) physiologic IOP load boundary (elastic material), (**c**) idealized IOP load boundary (viscoelastic material), and (**d**) physiologic IOP load boundary (viscoelastic material) at three different IOPs.

**Figure 6:**
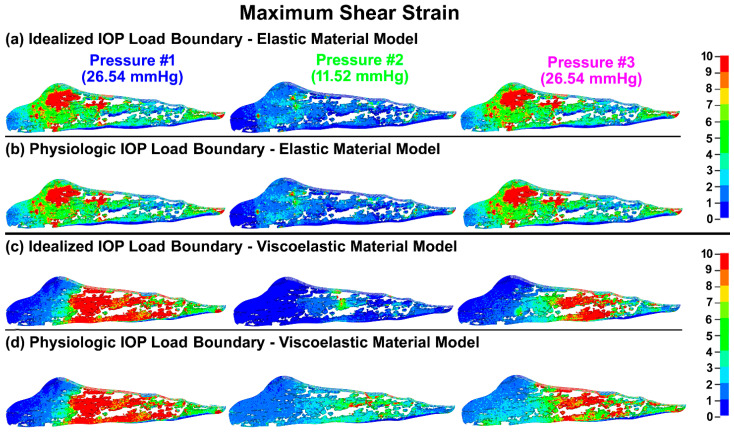
The maximum shear strain in the TM/JCT/SC complex with (**a**) idealized IOP load boundary (elastic material), (**b**) physiologic IOP load boundary (elastic material), (**c**) idealized IOP load boundary (viscoelastic material), and (**d**) physiologic IOP load boundary (viscoelastic material) at three different IOPs.

The maximum shear stresses and strains in the TM/JCT/SC complex with idealized and physiologic IOP load boundary and elastic and viscoelastic material models at three different pressures are shown in Figure 5 and Figure 6, respectively.

**Figure 7:**
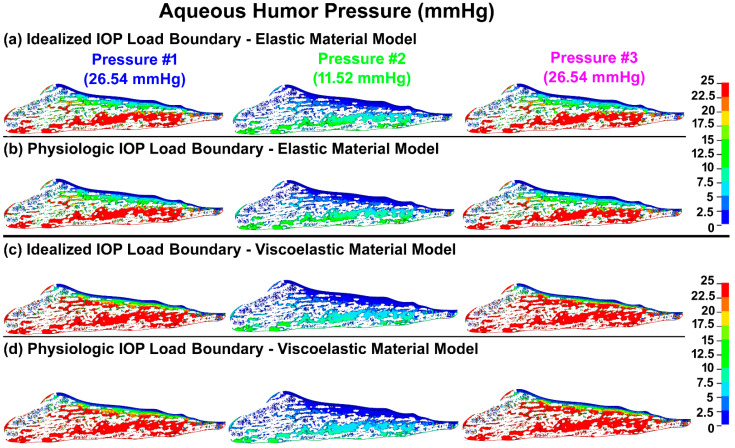
The pressure in the aqueous humor across the TM/JCT/SC complex with (**a**) idealized IOP load boundary (elastic material), (**b**) physiologic IOP load boundary (elastic material), (**c**) idealized IOP load boundary (viscoelastic material), and (**d**) physiologic IOP load boundary (viscoelastic material) at three different IOPs.

The pressure in the aqueous humor across the TM/JCT/SC complex with idealized and physiologic IOP load boundary and elastic and viscoelastic material models at three different pressures is shown in Figure 7.

**Figure 8:**
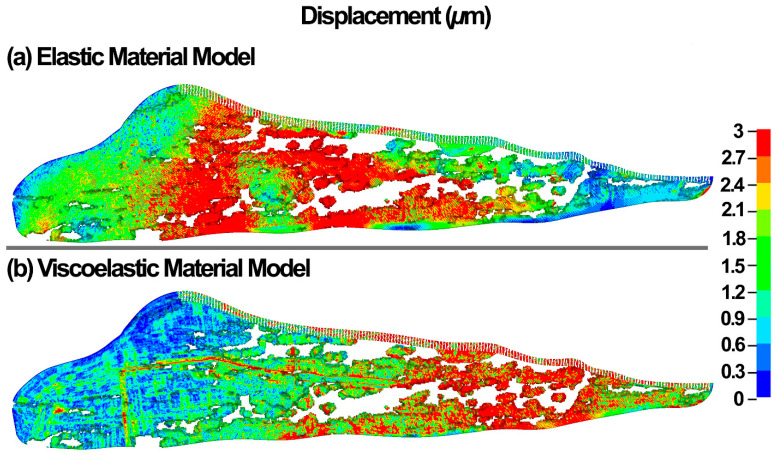
The displacement in the TM/JCT/SC complex with (**a**) elastic and (**b**) viscoelastic material model at the IOP of 10 mmHg.

The resultant displacement in the TM/JCT/SC complex at the IOP of 10 mmHg is shown in Figure 8.

The volumetric average stresses and strains in the TM/JCT/SC complex, as well as the average volumetric pressure in the aqueous humor across the TM/JCT/SC complex, are summarized in Table 1.

## 4. Discussion

Characterizing the mechanical behavior of the outflow tissues with a dynamic IOP load boundary may significantly contribute to our understanding of IOP regulation in the human eye [17]. Aqueous outflow resistance in the conventional outflow pathway is the predominant parameter in providing a balance between the average rate of aqueous inflow and outflow to maintain IOP within the normal physiologic range [59]. Experimental [59,74,101,102,103,104,105] and numerical [106,107,108] studies to date, and reviews of their findings [9,10,17,109,110,111,112,113,114,115], have all contributed greatly to our understanding of the mechanism of outflow resistance in the conventional outflow pathway. However, the active biomechanical response of the outflow tissues and their interaction with aqueous humor outflow dynamics have not been determined. The outflow pathway pressure provides a very dynamic mechanical environment that actively affects the tissues’ geometry [59,116] and causes a time-dependent pressure gradient across the outflow tissues [9,29]. Outflow resistance is affected by alterations in tissue geometry [117], so there must be a coupling between outflow hydrodynamics (fluid) and the biomechanics of the TM, JCT, and SC inner wall (structure) in the form of a fluid–structure interaction [58]. This coupling has been proven through experimental studies showing a correlation between the biomechanics of the outflow tissues and the rate of the aqueous outflow [57,59], suggesting that the outflow resistance actively contributes to aqueous outflow regulation to maintain an IOP within the normal range. In this study, a 3D FE microstructural model of the human TM/JCT/SC complex was constructed [60] (Figure 1) and subjected to an aqueous inflow with an idealized (steady flow) and physiologic IOP (pulsatile flow) load boundary (Figure 2). Outflow tissues were treated as elastic and viscoelastic. The material parameters were obtained from our prior study using an FE-optimization algorithm for healthy eyes matched with PhS-OCT imaging data [53].

Elastic materials behave the same regardless of time and loading rate, so the final resultant strain will be the same when load rate or time change [118]. As such, the first principal (tensile) stresses and strains in the TM/JCT/SC complex model with the elastic material model were similar regardless of the rate of the applied IOP load (Figure 3 and Figure 4; Table 1). The viscoelastic FE model showed larger stresses but smaller strains across the TM/JCT/SC complex than the elastic model (Figure 3 and Figure 4; Table 1). Transient IOP fluctuations cause a relatively large IOP difference (~20 mmHg) in a very short time frame (~0.1 s) (Figure 2a) that may result in viscoelastic rate stiffening in the outflow tissues. Rate stiffening reduces strains and causes a rate-dependent outflow pressure gradient across the outflow tissues, as observed in our modeling results (Figure 4 and Table 1). In the elastic TM/JCT/SC complex FE model, the strain appears immediately once the IOP load boundary is applied to the tissues. The strain is larger (Figure 3 and Figure 4; Table 1) and proportional to the applied IOP load boundary, which is different than the results from the viscoelastic model (Table 1). The viscous component of the viscoelastic material deforms slowly when exposed to an external force. Once a deforming force has been removed, the elastic portion of the material returns the tissue to its original configuration [119]. Viscoelastic materials present a reversible response depending on the rate of the applied load as they can return to the initial state [120,121,122], and this is why the resultant stresses and strains across the outflow connective tissues are IOP load rate-dependent (Figure 3 and Figure 4; Table 1).

TM biomechanics appears to be a key regulator of mechanosensing within the conventional outflow pathway [108]. When the TM expands with IOP elevation, it stretches the TM lamellae [2,29,57,123,124] and induces shear stress (Figure 5) and strain (Figure 6) in the SC endothelial cells due to circumferential flow through a narrowing SC lumen [125,126]. Calculating the shear stress of a viscoelastic outflow tissue with a physiologic IOP load boundary may significantly contribute to our understanding of IOP regulation through the endothelial nitric oxide pathway [67,68,69]. Viscoelastic outflow tissues showed a larger shear strain than elastic tissues (Figure 6 and Table 1). Shear strain in the viscoelastic FE model at pressure #1 was larger than in pressure #3 (Figure 6 and Table 1). While the magnitude of the pressure at these two points was the same at 26.5 mmHg, the IOP history was different (Figure 2b), which affects the tissue’s shear strain. In response to a shear strain, SC cells actively upregulate nitric oxide production, relaxing neighboring TM cells and increasing the permeability of the SC inner wall [67,68,69].

The TM/JCT interface and the inner wall of SC as well as distal to the outer wall of SC are predominantly responsible for ~50–75% and ~25–50% of the outflow resistance, respectively [58,62,127,128,129,130]. While the outflow resistance in the elastic FE model mainly resides within the TM/JCT, in the viscoelastic model, the JCT and the immediate vicinity of the SC inner wall are the main site of the outflow resistance (Figure 7). Aqueous outflow resistance results in a pressure gradient [9,30] in the outflow tissues that impacts the outflow system homeostasis and contributes to maintaining IOP in the normal range [131,132]. The pressure gradient also changes the outflow tissues’ geometry [25,26,27], which in turn affects the loading regime across the tissues, as well as the outflow resistance [28].

Li and colleagues showed that at a constant ocular pulse amplitude, the resultant TM displacement is IOP magnitude-dependent, whereby a larger IOP causes smaller displacement in the TM [33]. The smaller displacement suggests that the TM tissue must be viscoelastic. Li showed the average TM displacement of ~1.6 µm at an IOP of 10 mmHg [33]. In our study, the elastic and viscoelastic models resulted in the nodal-averaged TM displacements of ~2.0 and 1.5 µm at an IOP of 10 mmHg (Figure 8). Li [33] used nonhuman primate eyes (*Macaca nemestrina*) and we modeled human eyes, so the difference in TM displacements we report could be due to species-related differences in biomechanical tissue properties.

### Limitations

First, the geometries of the JCT and SC inner wall were considered as part of the segmented, reconstructed TM FE microstructure. However, we did not have any eye-specific dimensions for the JCT and SC inner wall, so they were reconstructed based on their average thicknesses from the literature: ~10 μm [71] and ~5 μm [30] for the JCT and SC inner wall, respectively. In addition, the µm-sized pores in the SC inner wall were not eye-specific and were distributed based on the data available in the literature with the density and size of 835 pores/mm^2^ [30] and 1.3 µm [75], respectively. While this study assumes pores are not artifactual, some may have different opinions and say pores are artifacts due to fixation. It has been shown that pore density in the SC inner wall decreases with reduced fixation time [30,75,112,133]. However, the in vivo existence of SC inner wall pores may need further clarification [112], which is outside of the scope of this study. Outflow is segmental with high- and low-flow regions, and one proposed route for the aqueous humor drainage across the inner wall is through giant vacuoles. Studies hypothesize that aqueous then passes through intercellular and intracellular pores [134]. An alternative to pores as a mechanism of aqueous passage from the JCT region to SC has been documented in multiple studies [17,135,136]. Funnel-like conduits arise from the SC inner wall endothelium and cross the SC to attach to the external wall. These aqueous flow conduits connect the TM and distal pathways, leading to TM and distal pathway synchronous motion [17]. Evidence suggests that modeling flow pathways distal to the TM would be valuable, but that is outside the scope of the current study.

Second, the same mechanical properties were used for the TM, JCT, and SC inner wall, but they have different properties. Although the differences could be a limitation of this study, the viscoelastic mechanical properties of the TM, JCT, and SC inner wall as separate tissues are still unknown. In a future study, we will calculate the mechanical properties of the TM, JCT, and SC inner wall separately using the FE-optimization algorithm matching with PhS-OCT imaging data.

Third, only one IOP cycle was simulated herein; the resultant stresses and strains may show larger differences if the simulation is performed in several loading cycles. In future studies, we will attempt to perform simulations with several IOP cycles.

Finally, this pilot study explored the feasibility of characterizing the viscoelasticity and dynamic IOP relationships and their resultant stresses and strains. Future studies will benefit from a larger cohort of healthy eyes to better generalize the tissues’ accurate geometry and biomechanical responses.

## 5. Conclusions

A 3D eye-specific elastic and viscoelastic microstructural FE model of a healthy human outflow pathway was established and subjected to both idealized and physiologic IOP load boundaries. This model allowed us to calculate the stresses and strains in the outflow tissues and the hydrodynamics of the aqueous humor. The results revealed that while the viscoelastic material model includes the IOP load rate in the resultant stresses and strains across the outflow pathway, the elastic material model results in the same stresses and strains regardless of the IOP load rate. The outflow pathway is subjected to a very dynamic, physiologic IOP load boundary and these model results suggest that the parameters should include and consider the applied IOP load rate to better estimate the stresses and strains in the outflow tissues and the hydrodynamics of the aqueous humor. The improved understanding may contribute to our knowledge of aqueous outflow dynamic regulation in the conventional outflow pathway.

## Figures and Tables

**Table 1:** Volumetric average stresses and strains in the TM/JCT/SC complex and volumetric average pressure in the aqueous humor.

Simulations	1st Principal Stress (kPa)	1st Principal Strain (%)	Max Shear Stress (kPa)	Max Shear Strain (%)	AH Pressure (mmHg)
**Idealized IOP Load Boundary—Elastic Material Model**					
Pressure #1	−3.18	4.83	0.68	4.55	13.88
Pressure #2	−1.17	1.22	0.35	1.95	4.02
Pressure #3	−3.17	4.83	0.69	4.55	13.88
**Physiologic IOP Load Boundary—Elastic Material Model**					
Pressure #1	−3.18	4.83	0.68	4.55	13.88
Pressure #2	−1.17	1.22	0.35	1.95	4.02
Pressure #3	−3.17	4.83	0.69	4.54	13.88
**Idealized IOP Load Boundary—Viscoelastic Material Model**					
Pressure #1	−4.95	2.15	9.84	7.66	12.01
Pressure #2	−3.12	0.92	4.12	2.29	3.98
Pressure #3	−6.12	2.22	10.65	4.55	11.11
**Physiologic IOP Load Boundary—Viscoelastic Material Model**					
Pressure #1	−4.95	2.15	9.84	7.66	12.01
Pressure #2	−2.99	0.79	4.75	2.93	4.35
Pressure #3	−5.41	2.33	11.59	3.89	11.42

## Data Availability

The raw/processed data required to reproduce these findings cannot be shared at this time as the data is part of an ongoing study.
